# Dropsy of the Antrum

**Published:** 1883-07

**Authors:** A. D. Blackader


### DROPSY OF THE ANTRUM.

Richard W., aged 10, was first brought to me for advice in the beginning of October, 1881, in reference to a fullness of the left cheek and prominence of the malar process on that side. He was a delicate-looking lad, belonging to a somewhat strumous family, but had enjoyed fair health up to the present. During the previous winter he had been hit by a hard snowball on that cheek, of the effects of which he had complained for some time; and early in the spring he had received another severe blow on the bridge of his nose, which his mother at the time thought had dislocated the nasal bones. I saw him, however, on the next day, and could not then make out any displacement.

His mother referred the present trouble to these injuries. The fullness had been first noted some six weeks previously, and had been gradually increasing since. At the time of her visit it was very evident on inspection. On examination there was found to be marked bulging of all the anterior wall of the antrum. The child was in good health otherwise. There was no difficulty in breathing through the nose, nor any sign of tumor in the pharynx or nasal cavities. I saw him at intervals during the next six weeks, during which the fullness slowly increased, but no softening of the bone was discovered. I now asked Dr. Roddick to see the case with me. He agreed with me in regarding it as due to pressure from within the antrum. On careful examination this time, now eight weeks after date of first consulting me, limited softening and slight fluctuations could be detected on the root of the second bicuspid tooth. His health otherwise remained good. The following week fluctuation was quite evident over the roots of the canine and the two bicuspids, and crackling of the thinned bone was felt distinctly. A puncture with a scalpel was made over the root of the first bicuspid, and about ʒij of glairy, colorless fluid escaped, with relief to the local tension. This again shortly accumulated, and five days afterwards the whole facial surface of the bone was pliable, crackling under pressure, and the hard palate on that side for about half an inch back from the alveolar process was bulging.

The lad was taken to the office of Mr. Alf. Wright, dentist, who carefully removed the canine and two bicuspids on the affected side. Their fangs were almost absorbed. The alveolar process was soft and crumbling, so that care had to be taken to injure it as little as possible. With the removal of the teeth a large quantity of glairy fluid escaped from the antrum through the opening thus made into its floor. Mr. Wright afterwards made a plate supporting a hard rubber tube, about the diameter of a goose quill, which passed into the cavity through the opening made by the first bicuspid, and kept it drained. A lotion of carbolized water was also given to the mother with directions to have the cavity syringed out frequently. With the removal of the pressure the prominence at once disappeared, reducing completely the deformity of the face. The cavity filled up, and now after the lapse of eighteen months there is no appreciable difference between the two sides.

The diagnosis in this case was somewhat difficult at first, and a more formidable tumor of fibrous or myxomatous character at first feared. The nature of the case was, however, made evident by the first puncture. The injuries the child received probably had a good deal to do with the formation of the cyst, but whether by closing up the aperture connecting the antrum with the nasal cavity, or whether by inducing some morbid change in the mucous glands, naturally existing in the lining membrane of the antrum, it is difficult to say.

After the operation, Dr. Roddick, who was present and whose advice was taken throughout the case, and myself, endeavored to make out the position of this aperture in the nostril, but both of us failed.

A very similar case is quoted by Mr. Pollock in Holmes' Surgery, where the cyst occupied a large portion of the left side of the upper jaw. Its anterior wall bulged out the side of the face. A free incision was made into the cyst from within the cheek. The anterior wall was found to be partly membranous, and partly consisted of thin flakes of bone. A portion was readily removed, so that a free opening was left for the escape of the contents, which were of a glutinous consistence and brownish in color. At the bottom of the cyst there was found projecting into its cavity the extreme point of the fang of the canine tooth. On its removal the fang was found partially necrosed. The cyst filled and closed in a short time without further treatment. The patient was about ten years of age, and when he quitted the hospital there was no trace of disease nor the slightest disfigurement.—Dr. A. D. Blackader *in Canada Medical and Surgical Journal.*

